# “Does the *Salmonella* Genomic Island 1 (SGI1) confer invasiveness properties to human isolates?”

**DOI:** 10.1186/s12879-017-2847-1

**Published:** 2017-12-01

**Authors:** Claire de Curraize, Lucie Amoureux, Julien Bador, Angélique Chapuis, Eliane Siebor, Claire Clément, Juliette Sauge, Ludwig-Serge Aho-Glélé, Catherine Neuwirth

**Affiliations:** 1 Bacteriology Department, University Hospital Dijon and UMR 6249, PTB, BP 37013, 21070 Dijon Cedex, France; 2 Epidemiology and Infection Control Department, University Hospital Dijon, Dijon, France

**Keywords:** *Salmonella enterica*, *Salmonella* Genomic Island 1 (SGI1), Invasiveness, Human, Antimicrobial resistance

## Abstract

**Background:**

In the eighties, a multidrug resistant clone of *Salmonella* Typhimurium DT104 emerged in UK and disseminated worldwide. This clone harbored a *Salmonella* genomic island 1 (SGI1) that consists of a backbone and a multidrug resistant region encoding for penta-resistance (ampicillin, chloramphenicol/florfenicol, streptomycin/spectinomycin, sulphonamides and tetracycline (ACSSuT)). Several authors suggested that SGI1 might have a potential role in enhancement of virulence properties of *Salmonella enterica*. The aim of this study was to investigate whether nontyphoidal *S. enterica* isolates carrying SGI1 cause more severe illness than SGI1 free ones in humans.

**Methods:**

From 2011 to 2016, all patients infected with nontyphoidal *S. enterica* in our hospital were retrospectively included. All nontyphoidal *S. enterica* isolates preserved in our University Hospital (Dijon, France) were screened for the presence of SGI1. Clinical and biological data of patients were retrospectively collected to evaluate illness severity. Statistical analysis of data was performed by Kruskal-Wallis test or Fisher’s exact test for univariate analysis, and by logistic regression for multivariate analysis.

**Results:**

A total of 100 isolates of *S. enterica* (22 serovars) were collected. Twelve isolates (12%) belonging to 4 serovars harbored SGI1: *S*. Typhimurium, *S*. Infantis, *S*. Kentucky, *S.* St Paul. The severity of the disease was age-related (for invasive infection, sepsis and inflammatory response) and was associated with immunosuppression (for invasive infection, sepsis and bacteremia) but not with the presence of SGI1 or with antimicrobial resistance.

**Conclusion:**

A rather high proportion (12%) of human clinical isolates belonging to various serovars (for the first time serovar St Paul) and harboring various antimicrobial resistance profile carried SGI1. Diseases due to SGI1-positive *S. enterica* or to antimicrobial resistant isolates were not more severe than the others. This first clinical observation should be confirmed by a multicenter and prospective study.

**Electronic supplementary material:**

The online version of this article (doi: 10.1186/s12879-017-2847-1) contains supplementary material, which is available to authorized users.

## Background

Nontyphoidal salmonellosis is a major zoonotic disease that most often causes gastroenteritis, but invasive infections can also occur and can be severe and life-threatening [1]. The elderly, infants and immunocompromised patients are more likely to have a severe illness. It has been pointed out that strains belonging to some serovars (Heidelberg or Dublin) or to some multidrug resistant epidemic clones such as the widespread *Salmonella* Typhimurium DT104 are probably more virulent [1]. A few in vitro studies suggest that the virulence in this penta-resistant clone is linked to the presence of a 43 kb integrative mobilizable element called *Salmonella* genomic island 1 (SGI1) [2, 3]. This element is made of a backbone and a multidrug resistance (MDR) region containing a cluster of genes responsible for the penta-resistance profile (ampicillin, chloramphenicol/florfenicol, streptomycin/spectinomycin, sulphonamides and tetracycline (ACSSuT)) [4]. SGI1 has been reported in various serovars including serovars Agona, Infantis, Kentucky, Paratyphi B variant Java, and so on [5]. Moreover many variants (SGI1-A to Z), in particular variants of the MDR region with other resistance genes, have been described, as well as a related genomic island, called *Proteus* genomic island 1 (PGI1) [5–13]. PGI1 has been notably reported in *S*. Heidelberg [13]. SGI1 is usually integrated at the 3’end of the *thdF* chromosomal gene of *Salmonella enterica* but it is sometimes inserted in a secondary chromosomal attachment site at the 3′ end of the *sodB* gene [14]. The prevalence of SGI1 in *S. enterica* is still unknown. Indeed, several studies have searched for the presence of SGI1 in collections of *S. enterica* strains selected according to their antimicrobial resistance or the presence of a class 1 integron or the serovar and therefore probably underestimated the prevalence of SGI1 because some SGI1 variants have no class 1 integron or MDR region [15, 16]. A Dutch study searched for the presence of a class 1 integron in 114 human strains of *S*. *enterica* and then searched for SGI1 in strains carrying a class 1 integron. They detected 9/114 (7.9%) isolates with SGI1 [17]. Another study detected 17 SGI1-positive isolates out of 90 isolates (19%) selected by their penta-resistance and the presence of a class 1 integron among 1920 isolates from an international collection [18]. A French study detected 16 SGI1 out of 28 (57%) human isolates of *S*. Typhimurium [19]. During the epidemic period (1990–2004) of *S*. Typhimurium DT104 in Scotland, the median prevalence of isolates not containing SGI1, estimated using a Bayesian approach, was 4% in humans [20]. The observation of severity illness in humans or in animals infected with the penta-resistant *S.* Typhimurium DT104 suggests that this clone is potentially more virulent [21, 22]. Several authors observed that SGI1 has a potential role in virulence by modulating expression of *S. enterica* genes, which could lead to hyper invasiveness [2, 23]. Other authors suggested that antimicrobial resistance increased severe of salmonellosis. One study observed that people were more likely to die after penta-resistant *S*. Typhimurium infection than were those with a pansusceptible *S*. Typhimurium infection [24]. Two other studies showed that the hospitalization rate was higher in patients with a resistant *S.* Typhimurium [25, 26].

The present study has been driven by the observation in our hospital of 2 severe cases of invasive infections due to SGI1-positive *S.* Typhimurium DT104. A 15-year-old immunocompromised boy receiving corticoids and tacrolimus after hematopoietic stem-cells transplantation (acute leukemia) developed bacteremia. Despite a treatment with ceftriaxone 4 g/day during 21 days a septic arthritis of the shoulder occurred 15 days after stopping antibiotic. The second case was a 12-year-old immunocompetent boy who developed a collapsus after severe diarrhea and vomiting caused by *S.* Typhimurium DT104.

Here, a retrospective study was conducted in our hospital to investigate whether SGI1-positive *S. enterica* cause more severe illness than those without SGI1. A second objective was to establish whether antimicrobial resistance has an impact on severity of the illness.

## Methods

### Study design

From 2011 to 2016, all patients infected by nontyphoidal *S. enterica* in our hospital were retrospectively included. When the isolate was not preserved in our laboratory, the patient was excluded. Clinical and biological data were retrospectively collected in medical records: age, sex, immunocompetence status, presence of invasive *S. enterica* infection (focal infections at systemic sites and/or bacteremia), sepsis, inflammatory response (hyperleucocytosis or increased CRP), presence of leukocytes and blood in stool, intravenous rehydration required. Patients were considered immunocompromised if they had underlying malignancies, HIV, receiving immunosuppressive treatments or suffering from sickle cell disease.

### Identification of isolates and phage-typing

Isolates were identified by biochemical tests using Api20E, Biomerieux®. They were serotyped using the agglutination method with *Salmonella* poly and monovalent sera, Biorad® according to internationally standardized methods based on the Kauffman-White scheme*.* In the absence of corresponding sera in our laboratory, isolates were serotyped by the French National Reference Center *Salmonella.* For *S.* Typhimurium isolates, the phage-type DT104 was screened by PCR as previously described [27].

### Susceptibility to antimicrobial agents

Susceptibility to antimicrobial agents was tested using the disk diffusion method on Mueller-Hinton agar in accordance with the CASFM- EUCAST guidelines of the year when the *S. enterica* was isolated. Antimicrobial profiles (susceptible or resistant) were retrospectively collected. The following antimicrobial agents were tested: amoxicillin, amoxicillin-clavulanic acid, ticarcillin, ticarcillin-clavulanic acid, piperacillin, piperacillin-tazobactam, cefotaxime, ceftazidime, cefepime, aztreonam, imipenem, nalidixic acid, ofloxacin, chloramphenicol, kanamycin, spectinomycin, streptomycin, tobramycin, amikacin, gentamicin, sulphonamides, trimethoprim and doxycycline.

### Detection of SGI1

Each nontyphoidal *S. enterica* isolate was screened for the presence of SGI1 by PCR targeting S026. This gene is highly conserved. To date, only one SGI1 variant without S026 (SGI1-Z) has been reported [11]. This powerful method detects all genomic islands (SGI1 or PGI1) whatever their chromosomal location, as previously described [13, 28]. To screen for the S026 gene, a bacterial DNA template was prepared by the heat lysis of cells. PCR amplifications were carried out with 200 μM deoxynucleoside triphosphates, 1.5 mM MgCl_2_, 0.25 μM of each set of primers (S026 F and S026 R), 1 U of *Taq* DNA polymerase (ThermoScientific®) and 1 μL of bacterial DNA. PCR amplifications were performed in a T3 thermal cycler, as follows: initial denaturation at 94 °C for 10 min; 30 cycles of denaturation at 94 °C for 1 min, primer annealing at 50 °C for 1 min and extension at 72 °C for 1.5 min; and a final extension at 72 °C for 10 min (Additional file 1).

To determine the type of genomic island, two PCR amplifications were performed on the DNA template of each positive strain with a set of specific primers (S005 R- S010 F) to detect SGI1 and with a set of specific primers (C1596 R - C1594 outF2) to detect PGI1 (Additional file 1). PCR amplifications were carried out with 300 μM deoxynucleoside triphosphates, 1.5 mM MgCl_2_, 0.25 μM of each set of primers, 1 U of *Taq* DNA polymerase (ThermoScientific®) and 1 μL of bacterial DNA. PCR amplifications were performed in a Biometra T3 thermal cycler, as follows: initial denaturation at 94 °C for 10 min; 35 cycles of denaturation at 94 °C for 1 min, primer annealing at 50 °C for 1 min 30 and extension at 72 °C for 7 min; and a final extension at 72 °C for 10 min.

### Statistical analysis

- Univariate analysis:

Data were analyzed using a Kruskal-Wallis test to compare quantitative variables as medians for age or the length of hospital stay in days. Fisher’s exact test was used to compare qualitative data. *P*-values <0.05 were considered statistically significant.

- Multivariate analysis:

Relationships between the presence of SGI1 and illness severity or between the presence of antimicrobial resistance and illness severity or the determination of risk factors of invasive infections were modeled using a logistic multiple regression model. A robust variance estimator was used and linearity was checked using fractional polynomials. Goodness of fit of the logistic model was assessed through the Homer-Lemeshow statistic.

Statistical analysis was performed with Stat software (Stat Version14).

## Results

### Proportion and characteristics of SGI1-positive nontyphoidal S. Enterica

From 2011 to 2016, 122 patients were included. Twenty-two patients were excluded because their isolates were not preserved. Twenty-two different serovars were detected by serotyping among the 100 isolates. Twelve (12%) patients were infected by SGI1-positive nontyphoidal *S. enterica*. No isolates harbored PGI1. The SGI1-positive isolates belonged to 4 serovars: *S*. Typhimurium, *S*. Infantis, *S*. Kentucky and *S.* St Paul. The SGI1-positive isolates belonged mainly to serovar Typhimurium (7 isolates) (Table 1). Moreover, the presence of SGI1 was significantly associated to the phage type DT104 (*p* = 0.001) (Table 2). Likewise, there was a link between penta-resistance (ACSSuT) and the presence of SGI1 (*p* < 0.001): two thirds of penta-resistant isolates were SGI1-positive. Despite the fact that history of recent travel was mostly not mentioned in medical records, it has to be noticed that four patients just coming back from Morocco were infected by SGI1-positive isolate: 2 belonging to serovar St-Paul and 2 belonging to serovar Kentucky.

**Table 1:** Characteristics of the strains isolated from patients infected by nontyphoidal *S. enterica*

Serovars	SGI1-positive n (%)	SGI1-negative n (%)
*S.* Typhimurium (*n* = 36)	7 (19.4)	29 (80.6)
DT104 (*n* = 9)	5 (55.6)	4 (44.4)
Non DT104 (*n* = 27)	2 (7.4)	25 (92.6)
Non *S*. Typhimurium serovars (*n* = 64)	5 (7.8)	59 (92.2)
*S*. 4, [5], 12: i- (*n* = 19)	0	19 (100)
*S*. Enteritidis (*n* = 14)	0	14 (100)
*S*. Paratyphi B var. Java (*n* = 2)	0	2 (100)
*S.* Dublin (*n* = 5)	0	5 (100)
Other serovars^a^ (*n* = 24)	5^b^ (20.8)	19 (79.2)
Phenotypes^c^	SGI1-positive n (%)	SGI1-negative n (%)
Pansusceptible (*n* = 21)	0 (0)	21 (100)
Resistant ≥1 (*n* = 79)	12 (15.2)	67 (84.8)
Including penta-resistant (ACSSuT)^d^ (*n* = 9)	6 (67)	3 (33)

^a^Other serovars (*n* = 24): *S*. Ahmadi (*n* = 1). *S*. Braenderup (*n* = 1). *S*. Chester (*n* = 2). *S.* Eastbourne (*n* = 1). *S*. Eboko (*n* = 1). *S*. Hadar (*n* = 1). *S*. Hessarek (*n* = 2); *S*. Infantis (*n* = 2); *S*. Kedougou (*n* = 1); *S*. Kentucky (n = 2); *S*. Napoli (*n* = 1); *S*. Panama (*n* = 1); *S*. Rissen (*n* = 1); *S*. Schwarzengrund (*n* = 2); *S*. Singapore (*n* = 1); *S*. St Paul (*n* = 3); and a new variant: 6, 7, − (n = 1)

^b^Other serovars with SGI1 (*n* = 5): *S*. Infantis (n = 1), *S*. Kentucky (n = 2), *S*. St Paul (n = 2)

^c^Phenotype was determined by testing amoxicillin, amoxicillin – clavulanic acid, ceftriaxone, nalidixic acid, chloramphenicol, kanamycin, spectinomycin, streptomycin, sulphonamides, trimethoprim and doxycycline in accordance to the antibiomicrobial guidelines of CASFM- EUCAST

^d^ACSSuT: Amoxicillin, Chloramphenicol, Streptomycin, Sulphonamides and Tetracyclines

**Table 2:** SGI1 and severity of the illness: comparison of clinical and biological data^a^

Variables	SGI1-positive	SGI1-negative	p-value^c^
N	12	88	
Age (years)	13.9 (56.9)	18.4 (54.2)	*p* = 0.62
Sex ratio M/F	5/7	42/46	*p* = 0.77
Immunosuppression	2 (16.7)	15 (17.0)	*p* = 1.00
Invasive infection	2 (16.7)	13 (14.8)	*p* = 1.00
Bacteremia	2 (16.7)	10 (11.4)	*p* = 0.63
Sepsis	2 (16.7)	13 (14.8)	*p* = 1.00
Fever or hypothermia	5 (41.7)	53 (63.9)	*p* = 0.21
Missing^b^	0	5	
Inflammatory response	11 (100)	71 (96.0)	*p* = 1.00
Missing^b^	1	14	
Bloody stool	2 (16.7)	20 (23.5)	*p* = 0.73
Missing^b^	0	3	
Leukocytes in stool	2 (16.7)	36 (43.9)	*p* = 0.11
Missing^b^	0	6	
Intravenous hydration required	5 (45.5)	41 (65.1)	*p* = 0.31
Missing^b^	1	25	
Length of hospital stay in days	2.5 (5)	4 (6)	*p* = 0.39
Infection with Serovar Typhimurium	7 (58.3)	29 (33.0)	*p* = 0.11
DT104 phage type	5 (41.6)	4 (4.5)	*p* = *0.001*
Infection with *S. enterica* harboring at least 1 resistance	12 (100)	67 (76.1)	*p* = 0.07
Infection with *S. enterica* harboring penta – resistance (ACSSuT)^d^	6 (50)	3 (3.4)	*p* < *0.001*

^a^Data are presented as median (interquartile range) for age and for long out of stay in hospital days or number of patients n (%)

^b^Missing refers to data not available in medical records

^c^Fisher’s exact test and Kruskal-Wallis test were performed as appropriate and p-values <0.05 were considered statistically significant

^d^ACSSuT: Amoxicillin, Chloramphenicol, Streptomycin, Sulphonamides and Tetracyclines

Number italicized were statically significant

### SGI1 and severity of the illness

From the clinical point of view, infections due to SGI1-positive isolates were not more severe than those due to isolates without SGI1. The relationship between the presence of SGI1 and invasive infection, bacteremia and sepsis was not statistically significant (Table 2). There was also no difference after performing multivariate analysis adjusted for age, sex and immunocompetence status. Corresponding adjusted Odds Ratios (aOR) were: 1.35 (CI_95%_ 0.31–5.86) for invasive infection, 1.82 (CI_95%_ 0.39–8.46) for bacteremia, 1.28 (CI_95%_ 0.21–7.68) for sepsis.

### Impact of antimicrobial resistance on the severity of illness

The rate of isolates fully susceptible to all antimicrobial classes was 21% (Table 3). Concerning an eventual link between antimicrobial resistance and severity of the illness it was surprising to notice that patients infected with a resistant isolate (harboring SGI1 or not) developed significantly less bacteremia (7.6%) than those infected with pansusceptible *S. enterica* (28.6%; *p* = 0.02) (Table 4). This result was confirmed by multivariate analysis adjusted for age, sex and immunocompetence status [adjusted Odds ratio (aOR) = 0.10 (CI_95%_: 0.02–0.55)]. Invasive infections [aOR = 0.16 (CI_95%_: 0.03–0.72)], and sepsis [aOR = 0.20 (CI_95%_: 0.05–0.80)] were also significantly less frequent in patients infected with a resistant isolate.

**Table 3:** Epidemiology of antimicrobial resistance in nontyphoidal *S. enterica* strains ^a^

Antibiotics	Percentage of resistance
Amoxicillin	37
Amoxicillin –clavulanic acid	17
Cefotaxime	2
Nalidixic acid	9
Ofloxacin	9
Kanamycin	3
Streptomycin	49
Spectinomycin	13
Sulphonamides	52
Trimethoprim	9
Chloramphenicol	12
Doxycycline	61

^a^Antimicrobial susceptibility testing was determined in accordance to the antimicrobial guidelines of CASFM-EUCAST

**Table 4:** Antimicrobial resistance and severity of illness: comparison of clinical and biological data

Variables	Infection with pansusceptible isolate^a^ (*n* = 21)	Infection with isolate harboring at least 1 resistance^a^ (*n* = 79)	Univariate analysis *p*-value^b^
Age (years)	22.3 (53.7)	15.8 (54)	0.26
Sex ratio (H/F)	12/9	35/44	0.28
Immunosuppression	1 (4.8)	16 (20.3)	0.11
Invasive infection	6 (28.6)	9 (11.4)	0.08
Bacteremia	6 (28.6)	6 (7.6)	*0.02*
Sepsis	6 (28.6)	9 (11.4)	0.08
Inflammatory response	19 (100)	63 (95.5)	1.00
Missing^c^	2	13	
Fever or hypothermia	13 (65)	45 (60)	0.80
Missing^c^	1	4	

^a^Data are presented as median (interquartile range) for age or number of patients n (%)

^b^Fisher’s exact test and Kruskal-Wallis test were performed as appropriate and p-values <0.05 were considered statistically significant

^c^Missing refers to data not available in medical records

Number italicized were statically significant

### Risk factors to develop nontyphoidal *S. enterica* invasive infections

Patients developing invasive infection, sepsis and an inflammatory response were older than other patients (55.6 versus (vs) 12.7 years old, *p* = 0.03; 57.1 vs 12.7 years old, *p* = 0.01 and 24.3 vs 2.6 years old, *p* = 0.04, respectively) (Table 5). In our study, five patients had underlying malignancies: one pancreatic cancer, one diffuse large B-cell lymphoma, one kidney cancer and two acute myeloid leukemias. Nine patients were received immunosuppressive treatment: infliximab for a Crohn’s disease (one case); mesalazine for a Crohn’s disease (one case); corticoid therapy for ulcerative colitis (one case); or multiple sclerosis (one case) or back pain following spinal arthrodesis (one case), corticoids and mycophenolate for a kidney transplant (one case), ciclosporine, corticoids and mycophenolate for a kidney transplant (one case), tacrolimus and everolimus for a liver transplant (one case), mycophenolate for lupus (one case). One patient, who also had lung cancer, was receiving everolimus, ciclosporine and corticoids for a heart transplant. Two patients were suffering from sickle-cell disease. As expected, the 17 immunocompromised patients had significantly more invasive infection (41.2% vs 9.6%, *p* = 0.003), bacteremia (29.4% vs 8.4%, *p* = 0.03) and sepsis (35.3% vs 10.8%, *p* = 0.02) than immunocompetent patients (Table 6) [1]. They were also significantly older than immunocompetent patients (54.2 vs 9.7 years old, *p* = 0.02). This observation was confirmed by multivariate analysis, except for bacteremia (aOR = 4.17 (CI_95%_: 0.99–17.57)). Indeed, immunocompromised patients developed more invasive infections [aOR = 6.30 (CI_95%_: 1.62–24.46)] and sepsis [aOR = 3.79 (CI_95%_: 1.03–13.91] than immunocompetent patients (*n* = 83).

**Table 5:** Age of patients and severity of illness: comparison of clinical and biological data

Variables (n: number of patients)	Median age^a^	iqr^a^	*p*-value^b^
Invasive infection			
No (*n* = 85)	12.7	53.5	*p* = *0.03*
Yes (*n* = 15)	55.6	61.4	
Bacteremia			
No (*n* = 88)	15.5	53.6	*p* = 0.08
Yes (*n* = 12)	43.1	64.6	
Sepsis			
No (*n* = 85)	12.7	48.8	*p* = *0.01*
Yes (*n* = 15)	57.1	46.1	
Fever or hypothermia			
No (*n* = 37)	29	58	*p* = 0.24
Yes (*n* = 58)	9.1	51.7	
Inflammatory response			
No (*n* = 3)	2.6	1.7	*p* = *0.04*
Yes (*n* = 82)	24.3	56.6	

^a^Data are presented in years

^b^Kruskal - Wallis test was performed and p-values <0.05 were considered statistically significant

Number italicized were statically significant

**Table 6:** Host immunocompetence and severity of illness: comparison of clinical and biological data

Variables	Immmunosuppression^a^		*p*-value^c^
	Yes (*n* = 17)	No (*n* = 83)	
Age (years)	54.2 (35.7)	9.7 (54.4)	*p* = *0.02*
Invasive infection	7 (41.2)	8 (9.6)	*p* = *0.003*
Bacteremia	5 (29.4)	7 (8.4)	*p* = *0.03*
Sepsis	6 (35.3)	9 (10.8)	*p* = *0.02*
Fever or hypothermia	7 (50.0)	51 (63.0)	*p* = 0.39
Missing^b^	3	2	
Inflammatory response	16 (100)	66 (95.7)	*p* = 1.00
Missing^b^	1	14	

^a^Data are presented as median (interquartile range (IQR) for age or number of patients n (%)

^b^Missing refers to data not available in medical records

^c^Fisher’s exact test and Kruskal-Wallis test were performed as appropriate and *p*-values <0.05 were considered statistically significant

Number italicized were statically significant

## Discussion

Our study is the first to evaluate the prevalence of clinical isolates of *S. enterica* harboring SGI1 without selection criteria. In our work, we detected SGI1 among various serovars and for the first time in serovar St Paul from 2 patients with a recent history of travel in Morocco. We observed a relatively high rate of 12% of SGI1-positive isolates. This rate is similar to the rate in a Dutch study (7.6%) [17]. Other studies estimated higher rates, probably due to selection on the antimicrobial resistance of isolates. In an Australian study, Levings et al. reported eight SGI1-positive isolates (20.5%) among a collection of 39 clinical strains harboring at least one resistance [29]. Krauland et al.*,* observed 17 SGI1-positive isolates (19%) among 90 penta-resistant isolates with a class 1 integron from a collection of 1920 international isolates [18]. Although, two thirds of penta-resistant isolates were SGI1-positive in our study, our results demonstrated that the penta-resistance (ACSSuT) was not a specific and sensitive marker of SGI1 since only half of the isolates carrying SGI1 harbored this profile in our study. As previously described, most SGI1-positive isolates belonged to the serovar *S*. Typhimurium (13/17, 76.4%) [18]. Finally, we confirmed the strong link between SGI1-positive *S*. Typhimurium and the DT104 phage-type (*p* = 0.001). A French study reported that 138 of 143 isolates of SGI1-positive *S*. Typhimurium (96.5%) belonged to the DT104 phage-type [30].

The resistant profile of our isolates was slightly different from the French data reported in the European report of EFSA in 2014 [31]. In our study the rate of resistance to nalidixic acid was much lower (9% vs 30%) and rates of resistance to amidinopenicillin, cyclines and sulphonamides were higher (37% vs 29.1%, 61% vs 40% and 52% vs 38% respectively). From the clinical point of view some of our results confirmed previous observations: elderly patients and immunocompromised patients were more likely to develop invasive nontyphoidal *S. enterica* infections [1]. Surprisingly we observed that patients infected by nontyphoidal *S. enterica* harbouring at least one resistance developed less invasive nontyphoidal *S. enterica* infections than those infected by pansusceptible isolates. This is not in accords with previous reports which describe an excess of mortality in Danish population or increase in burden of illness in Canadian population infected by multidrug resistant *S*. Typhimurium [24, 25]. Nevertheless, in a review of the literature on studies using chicken, mice and nematodes, Andersson et al.*,* reported that antimicrobial resistance of *S.* Typhimurium has a fitness cost that is typically observed as a reduced bacterial growth rate [32]. This might explain our observation. Concerning the impact of the SGI1 in virulence, our results did not confirm in vitro studies published to date. In previous reports, it was observed that SGI1 induced hyper invasiveness after exposure to rumen protozoa, partly due to S013 [2, 33, 34]. Other authors showed that collagenase mediated cytopathic effect without repression by the SlyA protein in the presence of SGI1 [22, 35]. Moreover Sahu et al.*,* underlined that the MDR region of. *S.* Typhimurium DT104 plays a direct role in virulence against *Caenorhabditis elegans* [3]. Finally, Golding et al. observed that the presence of SGI1 modulated the expression of *Salmonella* genes [23]. To date no study has been conducted in humans. In our study, we did not observe any difference in severity of illness between patients infected by SGI1-positive nontyphoidal *S. enterica* and those infected by SGI1-negative isolates. However, our work is a preliminary study and it should be interesting to confirm this result in a larger study.

## Conclusions

In this study, a *Salmonella* genomic island 1 has been detected in 12% of human clinical isolates belonging to various serovars and harbouring various antimicrobial resistance profiles. It might reflect a wide distribution of SGI1 among humans and probably also in animal isolates. Diseases due to SGI1-positive *S. enterica* or to antimicrobial resistant isolates were not more severe than the others. This first clinical observation should be confirmed by a multicenter and prospective study.

## Additional file

Additional file 1: Primers used for the PCRs. (DOCX 14 kb)

## Abbreviations

ACSSuT: Ampicillin, chloramphenicol/florfenicol, streptomycin/spectinomycin, sulphonamides and tetracycline
aOR: Adjusted odds ratio
CASFM- EUCAST: Comité de l’Antibiogramme de la Société Française de Microbiologie – European Committee on antimicrobial susceptibility testing
EFSA: European food safety authority
MDR: multidrug resistant
SGI1: *Salmonella* Genomic Island 1
Vs: Versus

## References

[1] Crump JA, Sjölund-Karlsson M, Gordon MA, Parry CM (2015). Epidemiology, clinical presentation, laboratory diagnosis, antimicrobial resistance, and antimicrobial Management of Invasive *Salmonella* Infections. Clin Microbiol Rev.

[2] Carlson SA, Sharma VK, McCuddin ZP, Rasmussen MA, Franklin SK (2007). Involvement of a *Salmonella* Genomic Island 1 gene in the rumen protozoan-mediated enhancement of invasion for multiple-antibiotic-resistant *Salmonella enterica* Serovar Typhimurium. Infect Immun.

[3] Sahu SN, Anriany Y, Grim CJ, Kim S, Chang Z, Joseph SW, et al. Identification of virulence properties in *Salmonella* Typhimurium DT104 using *Caenorhabditis elegans.* PLoS One [Internet]. 2013;8. Available from: http://www.ncbi.nlm.nih.gov/pmc/articles/PMC3790755/ Accessed 11 Nov 2015]. 10.1371/journal.pone.0076673 PMC3790755 24124587

[4] Boyd D, Peters GA, Cloeckaert A, Boumedine KS, Chaslus-Dancla E, Imberechts H (2001). Complete nucleotide sequence of a 43-kilobase genomic island associated with the multidrug resistance region of *Salmonella enterica* serovar Typhimurium DT104 and its identification in phage type DT120 and serovar Agona. J Bacteriol.

[5] Hall RM (2010). *Salmonella* genomic islands and antibiotic resistance in *Salmonella enterica*. Future Microbiol.

[6] Wilson NL, Hall RM (2010). Unusual class 1 Integron configuration found in *Salmonella* Genomic Island 2 from *Salmonella enterica* Serovar Emek. Antimicrob Agents Chemother.

[7] Targant H, Doublet B, Aarestrup FM, Cloeckaert A, Madec J-Y (2010). IS*6100*-mediated genetic rearrangement within the complex class 1 integron In104 of the *Salmonella* genomic island 1. J Antimicrob Chemother.

[8] Bi S, Yan H, Chen M, Zhang Z, Shi L, Wang H (2011). New variant *Salmonella* genomic island 1-U in *Proteus mirabilis* clinical and food isolates from South China. J Antimicrob Chemother.

[9] Siebor E, Neuwirth C (2011). The new variant of *Salmonella* genomic island 1 (SGI1-V) from a *Proteus mirabilis* French clinical isolate harbours *bla*_VEB-6_ and *qnrA1* in the multiple antibiotic resistance region. J Antimicrob Chemother.

[10] Lei C-W, Zhang A-Y, Liu B-H, Wang H-N, Guan Z-B, Xu C-W (2014). Molecular characteristics of *Salmonella* Genomic Island 1 in *Proteus mirabilis* isolates from poultry farms in China. Antimicrob Agents Chemother.

[11] Lei C-W, Zhang A-Y, Liu B-H, Wang H-N, Yang L-Q, Guan Z-B (2015). Two novel *Salmonella* Genomic Island 1 variants in *Proteus mirabilis* isolates from swine farms in China. Antimicrob Agents Chemother.

[12] Qin S, Qi H, Zhang Q, Zhao D, Liu Z-Z, Tian H (2015). Emergence of extensively drug-resistant *Proteus mirabilis* harboring a conjugative NDM-1 plasmid and a novel *Salmonella* Genomic Island 1 variant, SGI1-Z. Antimicrob Agents Chemother.

[13] Siebor E, Neuwirth C (2014). *Proteus* genomic island 1 (PGI1), a new resistance genomic island from two *Proteus mirabilis* French clinical isolates. J Antimicrob Chemother.

[14] Doublet B, Golding GR, Mulvey MR, Cloeckaert A. Secondary chromosomal attachment site and tandem integration of the Mobilizable salmonella Genomic Island 1. PLoS One [Internet]. 2008 ;3. Available from: https://www.ncbi.nlm.nih.gov/pmc/articles/PMC2297512/. Accessed 15 Aug 2017. 10.1371/journal.pone.0002060 PMC2297512 18446190

[15] Doublet B, Praud K, Bertrand S, Collard J-M, Weill F-X, Cloeckaert A (2008). Novel insertion sequence- and Transposon-mediated genetic rearrangements in Genomic Island SGI1 of *Salmonella enterica* Serovar Kentucky. Antimicrob Agents Chemother.

[16] Schultz E, Haenni M, Mereghetti L, Siebor E, Neuwirth C, Madec J-Y (2015). Survey of multidrug resistance integrative mobilizable elements SGI1 and PGI1 in *Proteus mirabilis* in humans and dogs in France, 2010–13. J Antimicrob Chemother.

[17] Vo ATT, van Duijkeren E, Fluit AC, Wannet WJB, Verbruggen AJ, Maas HME (2006). Antibiotic resistance, integrons and *Salmonella* genomic island 1 among non-typhoidal *Salmonella* serovars in The Netherlands. Int J Antimicrob Agents.

[18] Krauland MG, Marsh JW, Paterson DL, Harrison LH (2009). Integron-mediated multidrug resistance in a global collection of Nontyphoidal *Salmonella enterica I*solates. Emerg Infect Dis.

[19] Bugarel M, Granier SA, Weill F-X, Fach P, Brisabois A (2011). A multiplex real-time PCR assay targeting virulence and resistance genes in *Salmonella enterica* serotype Typhimurium. BMC Microbiol.

[20] Mather AE, Denwood MJ, Haydon DT, Matthews L, Mellor DJ, Coia JE (2011). The prevalences of *Salmonella* Genomic Island 1 variants in human and animal *Salmonella* Typhimurium DT104 are distinguishable using a Bayesian approach. PLoS One.

[21] Wall PG, Morgan D, Lamden K, Ryan M, Griffin M, Threlfall EJ (1994). A case control study of infection with an epidemic strain of multiresistant *Salmonella* Typhimurium DT104 in England and Wales. Commun Dis Rep CDR Rev.

[22] Wu MT, Carlson SA, Meyerholz DK (2002). Cytopathic effects observed upon expression of a repressed collagenase gene present in *Salmonella* and related pathogens: mimicry of a cytotoxin from multiple antibiotic-resistant *Salmonella enterica* serotype Typhimurium phagetype DT104. Microb Pathog.

[23] Golding GR, Olson AB, Doublet B, Cloeckaert A, Christianson S, Graham MR (2007). The effect of the *Salmonella* genomic island 1 on in vitro global gene expression in *Salmonella enterica* serovar Typhimurium LT2. Microbes Infect.

[24] Helms M, Vastrup P, Gerner-Smidt P, Mølbak K (2002). Excess mortality associated with antimicrobial drug-resistant *Salmonella* Typhimurium. Emerg Infect Dis.

[25] Martin LJ, Fyfe M, Doré K, Buxton JA, Pollari F, Henry B (2004). Increased burden of illness associated with antimicrobial-resistant *Salmonella enterica* serotype Typhimurium infections. J Infect Dis.

[26] Varma JK, Greene KD, Ovitt J, Barrett TJ, Medalla F, Angulo FJ (2005). Hospitalization and Antimicrobial Resistance in *Salmonella* Outbreaks, 1984–2002. Emerg Infect Dis.

[27] Pritchett LC, Konkel ME, Gay JM, Besser TE (2000). Identification of DT104 and U302 phage types among *Salmonella enterica* serotype Typhimurium isolates by PCR. J Clin Microbiol.

[28] Siebor E, Neuwirth C (2013). Emergence of *Salmonella* genomic island 1 (SGI1) among *Proteus mirabilis* clinical isolates in Dijon. France J Antimicrob Chemother.

[29] Levings RS, Lightfoot D, Partridge SR, Hall RM, Djordjevic SP (2005). The Genomic Island SGI1, containing the multiple antibiotic resistance region of salmonella enterica Serovar Typhimurium DT104 or variants of it, is widely distributed in other S. Enterica Serovars. J Bacteriol.

[30] Targant H, Ponsin C, Brunet C, Doublet B, Cloeckaert A, Madec J-Y (2010). Characterization of resistance genes in multidrug-resistant *Salmonella enterica* serotype Typhimurium isolated from diseased cattle in France (2002 to 2007). Foodborne Pathog Dis.

[31] European Food Safety Authority. The European Union summary report on antimicrobial resistance in zoonotic and indicator bacteria from humans, animals and food in 2014. EFSA J. 2016 [Internet];14(2). Available from: doi: 10.2903/j.efsa.2016.4380. 10.2903/j.efsa.2018.5182 PMC7009656 32625816

[32] Andersson DI, Hughes D (2010). Antibiotic resistance and its cost: is it possible to reverse resistance?. Nat Rev Microbiol.

[33] Ogunleye AO, Carlson SA (2012). Emergence of an SGI1-bearing *Salmonella enterica* serotype Kentucky isolated from septic poultry in Nigeria. J Infect Dev Ctries.

[34] Rasmussen MA, Carlson SA, Franklin SK, McCuddin ZP, Wu MT, Sharma VK (2005). Exposure to rumen protozoa leads to enhancement of Pathogenicity of and invasion by multiple-antibiotic-resistant *Salmonella enterica* bearing SGI1. Infect Immun.

[35] Carlson SA, McCuddin ZP, Wu MT (2005). SlyA regulates the collagenase-mediated cytopathic phenotype in multiresistant *Salmonella*. Microb Pathog.

