# Supplementary material for: “Does the Salmonella Genomic Island 1 (SGI1) confer invasiveness properties to human isolates?”
Source: BMC Infect Dis. 2017 Dec 1;17:741. doi: 10.1186/s12879-017-2847-1 (PMC5709944; doi:10.1186/s12879-017-2847-1)
Supplement: Additional file 1: — Primers used for the PCRs. (DOCX 14 kb) [file 12879_2017_2847_MOESM1_ESM.docx]

**Supplementary material**

Additional file 1: Primers used for the PCRs

| Primers | Nucleotide sequence (5’- 3’) |
| --- | --- |
| S026 F | TGGCTACTGCGGAACAAC |
| S026 R | TACCTGACTGCCTTCTAG |
| S005 R | GTTTAACGATGCGGGATC |
| S010 F | AGCTGAATATAGACGCGC |
| C1596 R | TCCTTGCTATTGGCGAAC |
| C1594 outF2 | GCTGGTCGATTCTTCGAC |
